# How universal is preference for visual curvature? A systematic review and meta‐analysis

**DOI:** 10.1111/nyas.14919

**Published:** 2022-10-26

**Authors:** Erick G. Chuquichambi, Oshin Vartanian, Martin Skov, Guido B. Corradi, Marcos Nadal, Paul J. Silvia, Enric Munar

**Affiliations:** ^1^ Human Evolution and Cognition Group (EvoCog) University of the Balearic Islands Palma de Mallorca Spain; ^2^ Department of Psychology University of Toronto Toronto Ontario Canada; ^3^ Danish Research Centre for Magnetic Resonance Copenhagen University Hospital Hvidovre Hvidovre Denmark; ^4^ Decision Neuroscience Research Cluster Copenhagen Business School Frederiksberg Denmark; ^5^ Department of Psychology Faculty of Health University Camilo José Cela Madrid Spain; ^6^ Department of Psychology University of North Carolina at Greensboro Greensboro North Carolina USA

**Keywords:** contour, hedonic liking, preference, vision, visual curvature

## Abstract

Evidence dating back a century shows that humans are sensitive to and exhibit a preference for visual curvature. This effect has been observed in different age groups, human cultures, and primate species, suggesting that a preference for curvature could be universal. At the same time, several studies have found that preference for curvature is modulated by contextual and individual factors, casting doubt on this hypothesis. To resolve these conflicting findings, we conducted a systematic meta‐analysis of studies that have investigated the preference for visual curvature. Our meta‐analysis included 61 studies which provided 106 independent samples and 309 effect sizes. The results of a three‐level random effects model revealed a Hedges’ *g* of 0.39—consistent with a medium effect size. Further analyses revealed that preference for curvature is moderated by four factors: presentation time, stimulus type, expertise, and task. Together, our results suggest that preference for visual curvature is a reliable but not universal phenomenon and is influenced by factors other than perceptual information.

## Graphical abstract

Evidence shows that humans exhibit a preference for visual curvature and suggests that this preference could be universal. However, several studies have found that preference for curvature is modulated by contextual and individual factors. We conducted a systematic meta‐analysis to resolve these conflicting findings. Our model revealed a medium effect size for a preference for visual curvature, but it was moderated by presentation time, stimulus type, expertise, and task.

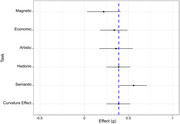

## INTRODUCTION

Contour is a core aspect of visual perception that plays a fundamental role in the detection and representation of objects.^1^ Contour integration binds disjointed parts of a scene into coherent global shapes and helps demarcate the interior of an object from its exterior.^2^, ^3^ The structure of object shapes is among the primary sources of information determining how objects are recognized.^4^ When asked to identify “what is this object?” people base their answers mainly on the shape and material properties of the perceived contour. In this sense, contour plays a critical role in how people perceive their surroundings and the objects within them.

Contour also informs how pleasing or displeasing objects are experienced to be. Early work dating back over a century sought to examine the effect that contour has on people's feelings.^5^, ^6^, ^7^ Those studies manipulated contour using stimuli, such as lines or abstract displays, and consistently showed that curvilinear forms are experienced as softer and more pleasant than angular forms, which are in turn experienced as harder and more serious. In other words, there was early recognition that contour can have an impact on the viewer's affective system, as reflected by Gordon's^8^ assessment that “curves are in general felt to be more beautiful than straight lines. They are more graceful and pliable, and avoid the harshness of some straight lines.” This early association between visual form and affect set the stage for examining the effect of contour on hedonic valuation.

More recently, a great number of studies have found that objects that exhibit curvilinear contour are preferred to objects that exhibit angular contour (for reviews, see Refs. 9 and 10). In addition to abstract and isolated forms and lines,^11^ this preference for curvilinear contours has been observed across a wide range of objects, including everyday artifacts and natural entities, building facades, interior rooms, as well as visual art.^12^, ^13^, ^14^, ^15^, ^16^

Because curvilinear forms appear to be more liked than angular forms irrespective of object category, it has been suggested that this preference has been selected for in the course of human evolution.^17^, ^18^ Bar and Neta^12^, ^19^ proposed that humans experience angular contour as unpleasant because this perceptual feature has become associated with threatening and dangerous objects. Curvilinear contour, they proposed, evokes feelings of pleasure, either because curvilinear objects signal an absence of danger, or because they have been associated with rewarding behavior throughout human evolution. In this sense, associating angularity with threat and curvilinear contour with reward can be seen as another example of “snap judgments” that people make about objects in their environment to maximize their chances of survival.

Over the last two decades, numerous behavioral and neuroscientific studies have tested this hypothesis. Unfortunately, evidence for and against it has been both mixed and inconclusive. Thus, some studies support the contention that human preference for curvature is universal, and, therefore, possibly innate.^20^ For example, Gómez‐Puerto and colleagues^21^ found that nonwestern participants in Mexico and Ghana prefer objects with curvilinear contour, as do participants in Spain. There is also mounting evidence that infants and children look longer at curvilinear than angular objects,^22^, ^23^, ^24^, ^25^, ^26^, ^27^, ^28^, ^29^ suggesting that evaluative responses to curved and angular objects may be present at birth. Finally, recent experiments have found evidence that chimpanzees and gorillas,^30^ as well as orangutans,^31^ also prefer objects that have curvilinear rather than angular contour. The observation of preference for curvature across different age groups, human cultures, and primate species is consistent with a possible universality of the effect.

Other experiments, however, have found compelling evidence that preference for curvature is influenced by both subjective sensitivity to contour features, as well as contextual factors. In two important studies, Corradi and colleagues^32^, ^33^ demonstrated that a group of participants who collectively exhibit a greater preference for curvilinear than angular stimuli also contains a nontrivial number of participants who do not share this general predilection. This finding suggests that some people's hedonic response to contour information differs from the majority's, implying that the observed preference pattern might not be universal after all. Possible sources of this variance include the kind and degree of exposure and knowing what the individual has been exposed to, among others. For example, people who have acquired an expertise‐level understanding of architectural design report a diminished liking for curvature combined with an enhanced liking for angular objects under certain conditions.^34^ Such evaluations may be particularly dependent on expertise because it supports the cognitive processing of the stimulus at different stages of the aesthetic experience.^35^ In addition, studies have also found that differences in personality^36^ and psychiatric conditions, such as autism spectrum disorder, can influence how individuals respond to stimuli with different contours.^37^, ^38^ Because persons with autism spectrum disorder exhibit a different constellation of emotional and perceptual processes compared to neurotypical controls, these findings suggest that preference for curvature is influenced by factors that vary across individuals due to the ways in which they might perceive and appraise objects in their environments, in a similar way to what has been observed in the case of symmetry.^39^

Contextual factors can also modulate the hedonic outcome of exposure to curvilinear and angular objects. Corradi and colleagues^40^ found participants’ propensity for choosing curvilinear over angular objects diminished when choices were not restrained by response time constraints. This suggests that preference for curvature emerges rapidly, and that its effect can be attenuated by top‐down processes that could exhibit themselves downstream in the processing pathway (e.g., semantics). Similarly, Palumbo and Bertamini^41^ collected two‐alternative forced‐choice responses (like vs. dislike) made during a fixed display window (i.e., 120 ms), and compared those with self‐paced continuous liking ratings, and found that preference for curvilinear objects was slightly more pronounced under the former than the latter condition. This too is consistent with the idea that the effect is stronger under conditions that favor quick, snap judgments. These authors also found that participants prefer curvilinear objects with a smaller number of vertices and a higher number of concavities when using the self‐paced rating scale as the evaluative anchor. Together, these results suggest that contextual conditions—including stimulus features, presentation time, and evaluative anchors—can affect the way in which contour information becomes evaluated, resulting in the assignment of different degrees of liking or disliking to objects with curvilinear and angular contour.

Finally, it remains unclear how the human brain computationally implements hedonic evaluations of curvature in the visual domain. The innateness hypothesis posits that representations of curvilinear contour engage neural processes associated with the generation of pleasure, while representations of angular contour engage neural systems involved in producing defensive emotional states such as fear. Using functional magnetic resonance imaging (fMRI), Bar and Neta^19^ found that angular objects elicit greater activity in the amygdala than curvilinear objects. They interpreted this result as potential evidence that exposure to angular objects produces a fear response that signals threat and danger. However, they found no evidence that pleasure‐related neural structures respond differently to curvilinear compared to angular objects. Vartanian and colleagues’^16^ fMRI experiment in the domain of architecture reported a different pattern of results to Bar and Neta's.^19^ Specifically, in relation to the neural activity associated with beauty judgments, Vartanian et al.^16^ found that rooms with curvilinear designs elicit greater activity in the anterior cingulate cortex (ACC) than rooms with angular designs. The ACC is a key structure within the neural system involved in the computation of core affect.^42^, ^43^ Furthermore, given its strong resting‐state connectivity with both the orbitofrontal cortex and the anterior insula, it is presumed to underlie emotional salience monitoring.^44^ Hence, the observed activation in the ACC could be explained as a difference in the amount of subjective pleasure experienced by the participants in response to the two categories of stimuli. Indeed, data collected outside of the fMRI scanner demonstrated that pleasantness ratings accounted for the majority of variance in beauty judgments. Yet, while this result suggests that curvilinear rooms might become liked by engaging appetitive affective processes, Vartanian and colleagues^16^ did not observe any difference in the modulation of amygdala activity by the two stimulus classes, or activation in regions of the brain that underlie the perception of visual features, including contour. Understanding the neurobiological bases of the evaluation of features such as contour may lie in charting the dynamics of the networks that integrate these regions.^45^

To make sense of this contradictory body of work, we conducted a systematic review and meta‐analysis of studies reporting hedonic evaluations of stimuli varying in contour in the visual domain. We aimed to assess the average effect size of preference for curvature across different stimulus types, experimental paradigms, contexts, and populations. This analysis had two goals: (1) ascertaining how universal liking for curvature truly is and (2) identifying factors that might moderate its effect size across different conditions.

## MATERIALS AND METHODS

### Protocol and registration

A preliminary protocol was made publicly accessible on the Open Science Framework (https://osf.io/58n23/) prior to data collection. The method for this systematic review and meta‐analysis was developed in line with the PRISMA‐P guidelines.^46^ The meta‐analysis examined studies comparing curvilinear (i.e., curved, smooth, round, and circular) and angular (i.e., rectilinear, straight, sharp‐angled, jagged, squared, pointed, and rectangular) visual stimuli in behavioral preference measures. Most of the studies on preference for curvature investigated the effect using continuous outcome measures. These measures provide independent mean values for each one of the groups compared (e.g., curvilinear vs. angular). In addition, a smaller set of studies investigated the effect using dependent dichotomous measures.^21^, ^30^, ^32^, ^40^, ^47^, ^48^, ^49^, ^50^, ^51^, ^52^, ^53^ These measures provide complementary preference values for each one of the groups compared. That is, in these studies, a curvilinear stimulus and an angular stimulus are presented simultaneously, and participants choose one of the two stimuli. Consequently, when participants select a stimulus 80% of the time, this also means that the other stimulus is selected 20% of the time, which indicates that the preference values are complementary to each other. Given the divergence between continuous and dependent dichotomous measures, the present analysis focused exclusively on continuous measures such as those using rating scales (e.g., liking) and two‐alternative responses (e.g., like‐dislike). However, although we did not include data from studies that employed dependent dichotomous outcomes or studies focusing on other measures, such as response times and eye movements in our meta‐analysis, they are nevertheless discussed throughout the meta‐analysis because they provide valuable insight into this effect.

### Eligibility criteria

The following criteria were established for eligible studies: (1) The study was empirical or experimental research published in a peer‐reviewed journal, it was presented as a doctoral dissertation, or it was presented at an international conference. Studies that focused on theoretical or conceptual aspects were excluded (e.g., Refs. 9 and 10). (2) Participants were human adults. Studies conducted with nonhuman samples (e.g., Refs. 30 and 31), infants or children (e.g., Refs. 25 and 54) were excluded. (3) The study was written in English. Studies written in other languages were excluded (e.g., Refs. 55 and 56). (4) The study was conducted with a neurotypical sample of participants. Studies that targeted clinical populations were excluded (e.g., the autism spectrum condition group from Palumbo et al.^37^). (5) The study compared curvilinear (i.e., curved, smooth, round, and circular) and angular (i.e., rectilinear, straight, sharp‐angled, jagged, squared, pointed, and rectangular) visual stimuli. Studies whose results focused on another sensory modality (e.g., Ref. 57) or did not include curvilinear or angular stimulus categories (e.g., Ref. 58) were excluded. (6) The measures of the study were based on personal preference. Measures based on reaction times (e.g., Refs. 59 and 60), and eye movement patterns or neurophysiological results (e.g., Ref. 24) were excluded. (7) The study employed a continuous outcome measure, such as a rating scale, or a two‐alternative procedure (e.g., like‐dislike and approach‐avoidance). Studies using dependent dichotomous outcome measures (e.g., Refs. 21 and 33) were excluded. No temporal constraint was settled for the year of publication of the study.

### Search strategies

The search of studies followed the strategies described in the protocol, and it was conducted on February 21, 2021. First, the search of studies was carried out via the electronic databases EBSCOHost—PsycINFO, PubMed, and Web of Science (WoS). We employed generic searches within the Title, Abstract, and Keywords using the following combination of terms: (curvature OR curvilinear OR smooth OR round OR curved), (sharp‐angled OR angular OR straight OR rectilinear), (contour OR shape), AND (aesthetics OR preference OR liking OR beauty). Second, the search of studies was carried out via journal searches within six relevant journals in the domain of empirical aesthetics: Psychology of the Aesthetics, Creativity, and The Arts; Empirical Studies of the Arts; Perception; i‐Perception; Acta Psychologica; and British Journal of Psychology. Lastly, after screening all the studies against eligibility criteria, manual “backward” and “forward” search from the citation and reference lists of the remaining studies was implemented via the Google Scholar search engine (see the Supplementary Information for additional details on the literature search process).

### Study selection

The initial search of studies provided 696 studies via database searching and 77 studies via journal searching. After the literature search, two authors independently screened the studies against inclusion criteria. In cases of discrepancies, a third author screened the studies again to reach an agreement among the authors. When a study did not report the necessary data to calculate all the effect sizes, the authors were contacted via e‐mail and asked whether it was possible to obtain the data to calculate an effect size. In cases of no response and when the studies represented relevant values in plots (i.e., means, confidence intervals, or standard errors), we used a web plot digitizer^61^ to convert plotted representations into numerical values. Conversely, when these studies had no available plots, they were excluded because of insufficient data for calculating effect sizes.

### Data extraction and management

Data were extracted in accordance with the PRISMA‐P guidelines.^62^ According to the protocol, the following basic information was extracted from the studies meeting eligibility criteria: (1) author/s name/s, (2) year of publication, (3) title of the study, (4) journal/conference name, (5) study design (within‐subjects vs. between‐subjects), (5) sample size, (6) number of male and female participants, (7) mean age and standard deviation, (8) mean and standard deviation (or standard error) values of curvilinear and angular preference. We identified wide variability among the concepts employed by researchers to measure preference for curvature in the visual domain. Therefore, deviating from the protocol, this variable was also extracted and analyzed along with the other possible moderator variables registered in the protocol: (9) task (i.e., the construct used to measure the curvature effect), (10) stimulus type, (11) presentation time of the stimuli, (12) measure (i.e., whether preference was measured using a rating scale or two‐alternative response options), and (13) expertise (e.g., architects, designers, art students, and laypeople). Additional variables considered for exploratory analyses were extracted and coded as follows: (14) data collection procedure (in‐person vs. online, paper‐based, computer‐based, web‐based, projection screen‐based, and tablet‐based), and (15) verbal terminology (e.g., curvilinear/rectilinear, round/angular, curved/sharp, curved/angular, round/sharp, circular/rectangular, etc.). Lastly, we also coded (16) the stimulus dimensionality (two‐dimensional vs. three‐dimensional) and (17) digitization (i.e., whether the study plots were digitized or not [only in cases of insufficient data to calculate effect sizes and if plots were available] to perform sensitivity analyses).

The variable *task* indicates the evaluative construct researchers asked participants to assess when responding to curvilinear and angular stimuli. It included terms, such as approach/avoidance, attractiveness, beauty, comfortableness, valence, liking, pleasantness, preference, price expectation, intention to purchase, and wanting, among others. Given the wide terminology employed by researchers, we categorized the terms into five distinct categories: artistic, semantic, economic, hedonic, and magnetic. The *artistic* task (*k* = 42) included the experiments using the concepts beauty, beautiful/ugly, and beauty/not beauty, which were typically used in studies of art objects. The *semantic* task (*k* = 35) included the experiments using the bipolar adjectives good/bad, positive/negative, dangerous/safe, safe/unsafe, fear/safety, aggressive/peaceful, hostile/friendly, threatening/protective, harsh/gentle, irritated/balanced, sad/cheerful, and comforting/not comforting. This categorization was based on the semantic differential scales of the evaluative dimension proposed by Osgood et al.^63^ to measure the value of an object. The *economic* dimension (*k* = 31) included the experiments where participants were instructed to indicate purchase likelihood, willingness to buy, willingness or intention to purchase, price expectation, and price to pay. The *hedonic* task (*k* = 184) included the experiments using the terms attractiveness, liking, pleasantness, preference, and appealing. Lastly, the *magnetic* task (*k* = 17) included experiments using attraction‐related terms, including approach, approach/avoidance, willingness to enter/exit, and wanting.

The variable *stimulus type* was coded using four levels: object (*k* = 123), meaningless (*k* = 83), spatial design (*k* = 73), and symbolic design (*k* = 30). Within the domain of empirical aesthetics, several studies have documented a preference for curvature using real objects as well as meaningless stimuli (e.g., Refs. 12, 19 and 64). Corradi and Munar also noted an increasing number of studies examining preference for curvature from applied research fields (i.e., advertising, marketing, packaging, and interior design). These authors described the stimuli from these studies as item forms, product packaging or logos, and general settings. Therefore, influenced by the review of Corradi and Munar,^10^ we also included as stimulus type the levels “spatial design” (e.g., interior designs, architectural façades, etc.) and “symbolic design” (e.g., logos, typefaces, etc.).

Regarding the variable *presentation time* (of the stimulus), it varied across studies from relatively brief presentation times (e.g., 84, 85, 90, 120, 500, 1500, 2000, 3000, and 7000 ms) to studies that allowed unlimited time for responding. However, we found that the number of effect sizes within each specific presentation time was small. Therefore, we coded this variable into two levels: limited (*k* = 50; i.e., from 84 to 7000 ms) and unlimited (*k* = 259, i.e., until response) presentation times for the stimuli. The variable measure indicates whether the task used by researchers was a continuous measure (*k* = 275; e.g., rating scale) or a continuous dichotomous measure (*k* = 34; e.g., like‐dislike two‐alternative forced choice procedure).

Finally, the variable *expertise* indicates whether a study included participants qualified as experts or quasi‐experts. Some examples include working architects and designers who were presented with images of architectural interior designs,^34^ orthodontists and restorative dentists presented with pictures of teeth,^65^ and university‐level design students presented with images of architectural interiors.^37^ We coded this variable in three levels: experts (*k* = 34), quasi‐experts (*k* = 8), and nonexperts (*k* = 267).

### Meta‐analytic strategy

Data analysis was carried out with the R environment for statistical computing,^66^ using the “metafor” package.^67^ We noted that several studies provided multiple effect sizes using the same sample of participants. A critical assumption in random‐ and mixed‐effects meta‐analyses is that the effect sizes are independent. When the effect sizes in a meta‐analysis are not independent, the estimated standard errors for the average effect are underestimated.^68^ Therefore, deviating from the protocol, we handled the dependencies between the effect sizes with a three‐level random effects meta‐analysis with restricted maximum likelihood.^69^, ^70^ The three‐level meta‐analysis model is an extension of the random‐effects meta‐analysis model.^71^ This model divides variability in effect sizes into the sampling variation for each effect size (Level 1), variation across multiple effect sizes within a study (Level 2), and variation across studies (Level 3).^72^, ^73^ Thus, compared to other approaches, a three‐level meta‐analysis allows researchers to study and decompose heterogeneity variances at different levels.

We conducted a study of influential cases based on Cook's distance (Di), which indicates the relative influence of each effect size on the summary estimate. As a standard rule of thumb, Di values greater than three times the mean Di were considered influential cases^74^ (17 effect sizes from 14 studies^32^, ^41^, ^48^, ^53^, ^64^, ^75^, ^76^, ^77^, ^78^, ^79^, ^80^, ^81^, ^82^, ^83^). In addition, two more sensitivity analyses were performed separated by repeating the models without the effect sizes extracted from the values represented in plots (19 effect sizes from eight studies^53^, ^76^, ^84^, ^85^, ^86^, ^87^, ^88^, ^89^), and without the effect sizes of studies using real (three‐dimensional) stimuli (26 effect sizes from 10 studies^75^, ^76^, ^79^, ^82^, ^84^, ^85^, ^87^, ^90^, ^91^, ^92^).

We fitted moderator models to evaluate how specific variables accounted for the variability among effect sizes. We considered as potential moderators of preference for curvature the variables *task* (i.e., artistic, economic, semantic, hedonic, and magnetic), *stimulus type* (meaningless, object, spatial design, and symbolic design), *presentation time* (limited vs. unlimited), *measure* (continuous vs. continuous dichotomous), and *expertise* (experts, quasi‐experts, and nonexperts). Lastly, exploratory analyses were also considered with the variables, including *year of publication*, *task modality* (in‐person vs. online, paper‐based, computer‐based, web‐based, projection screen‐based, and tablet‐based), and *verbal terminology* (e.g., curved/sharp, curvilinear/rectilinear, round/angular, etc.).

## RESULTS

We consider Hedges’ *g* for a 95% confidence interval as the main summary measure. Hedges’ *g* provides an unbiased estimate of the effect size because it does not overestimate the magnitude of the effect of studies with small sample sizes.^93^ We interpret values of 0.15, 0.40, and 0.70 as small, medium, and large effects, respectively.^94^ Unless otherwise indicated, all analyses were preregistered (https://osf.io/58n23/).

### Included effect sizes

**FIGURE 1:**
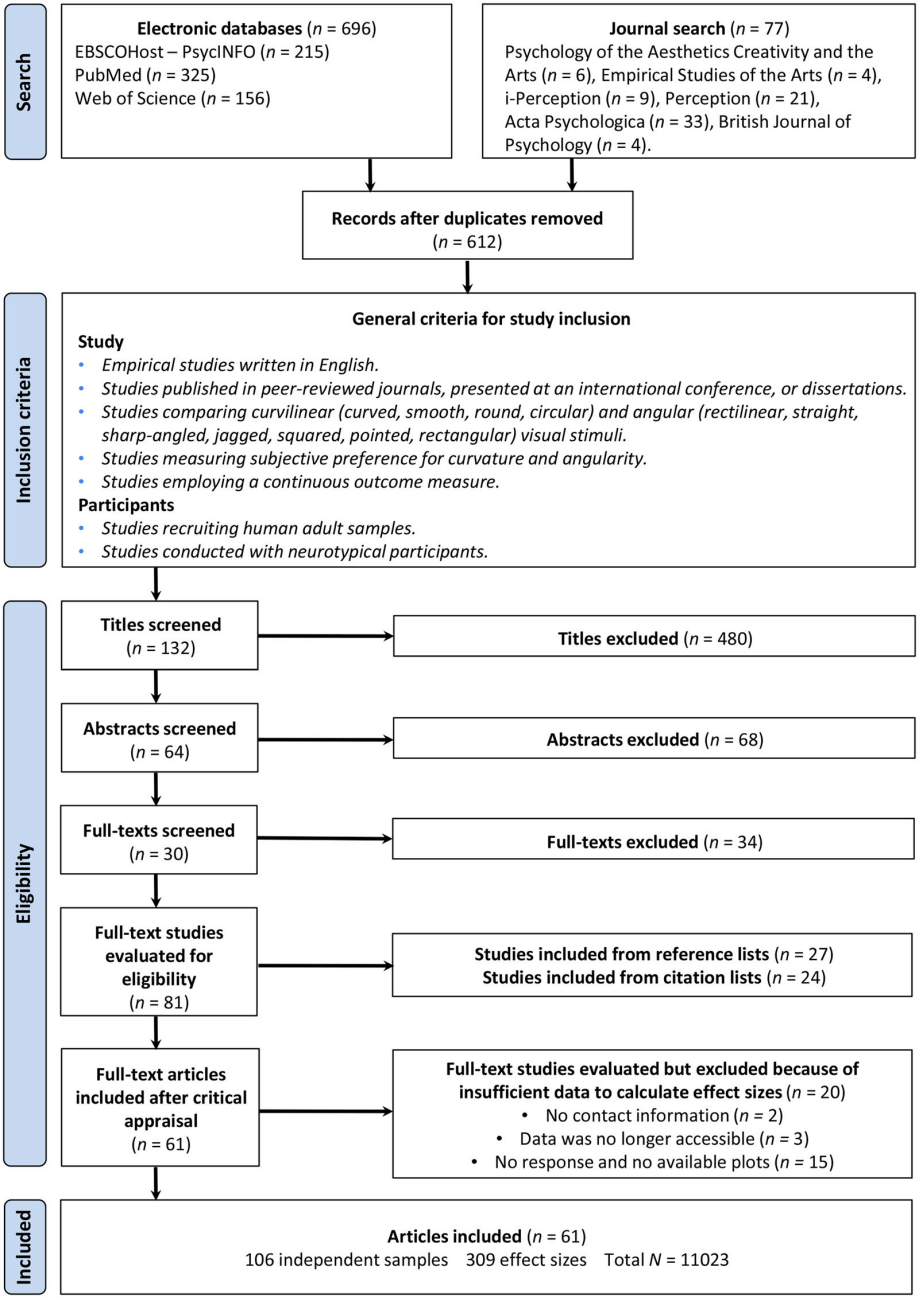
PRISMA flow diagram for the inclusion process. A total of 61 records fulfilled the eligibility criteria.

The initial search of studies provided 696 studies via database searching, and 77 studies via journal searching. The removal of duplicate studies left 612 studies to be screened for eligibility. Of those, 548 studies were excluded because their title and abstract did not fit the subject matter of the meta‐analysis. Full‐text screening of the 64 remaining studies resulted in the inclusion of 30 in the meta‐analysis. Manual “backward” and “forward” search from citation and reference lists of the remaining studies provided 27 additional studies from reference lists and 24 additional studies from citation lists. In total, 81 studies were identified for inclusion, providing a pool of 141 independent samples and 418 effect sizes. The process of gathering data from these studies revealed that 42 studies missed the data that were relevant to the calculation of effect sizes. The authors of 16 of these studies provided raw data when contacted, but two of these datasets were impossible to interpret. The authors of three other studies reported that the data were no longer accessible, the authors of 15 studies did not reply, and the authors of two studies could not be contacted because of a lack of viable contact information. Therefore, of the 42 studies with missing data, we were unable to retrieve data relevant to the calculation of effect sizes from 28 studies. Eight additional studies were included by obtaining the summary scores represented in reported plots.^53^, ^76^, ^84^, ^85^, ^86^, ^87^, ^88^, ^89^ Studies without available plots were excluded because of insufficient data. All in all, of the 81 studies meeting inclusion criteria, 61 studies were included in the final meta‐analysis. Figure 1 depicts a flowchart with detailed information on the literature search and the inclusion process.

**FIGURE 2:**
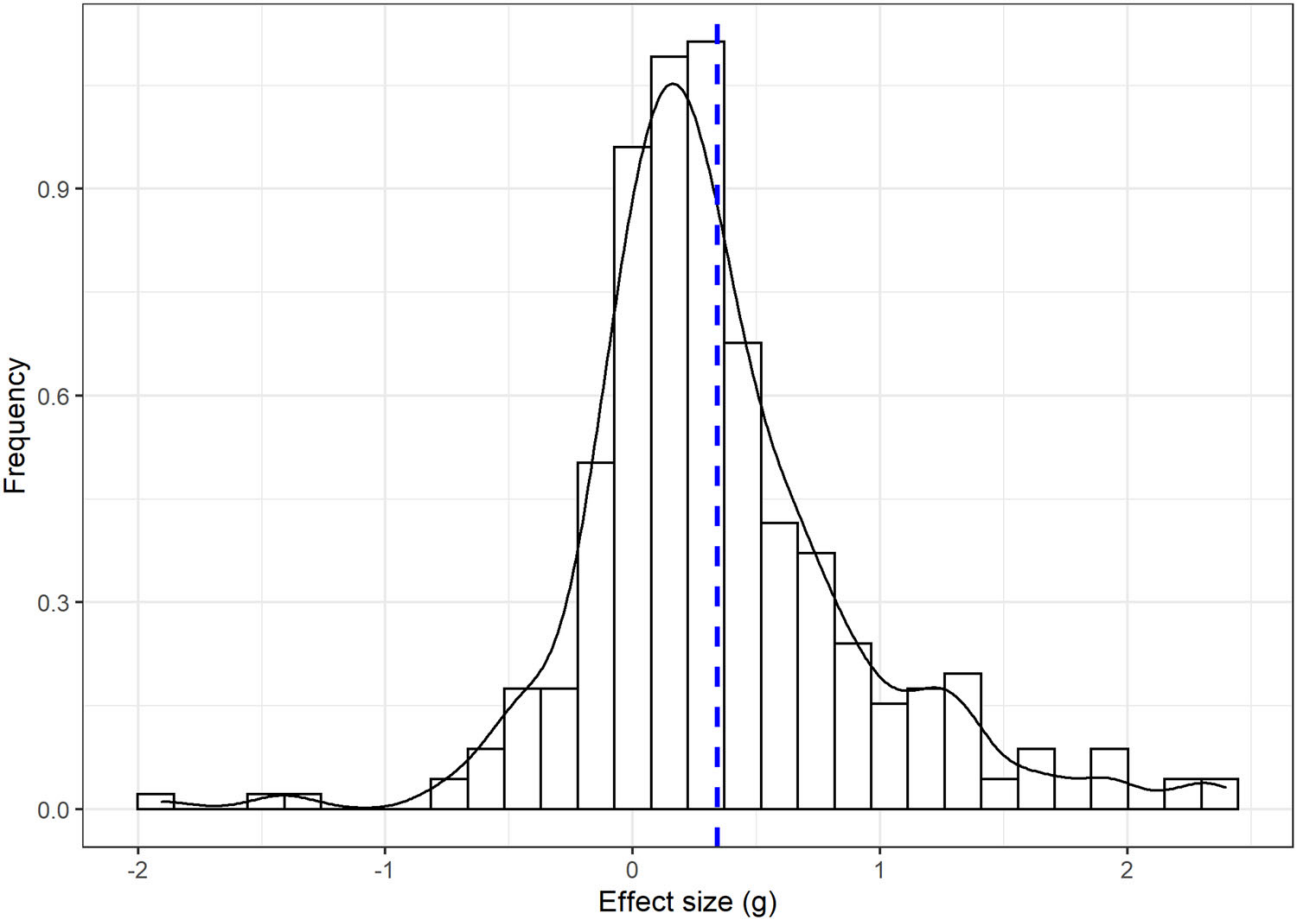
Histogram of the included effect sizes. The dashed line represents the mean value.

The 61 studies included in the analysis yielded 106 independent samples and 309 effect sizes (Figure 2). Statistics for the effect sizes and the samples of the studies are summarized in Table 1 (11,023 participants, M_age_ = 27.81, SD_age_ = 7.34).

**TABLE 1:** Summary statistics of the included studies

*N* studies = 61	Mean	Median	SD	Minimum	Maximum
Effect size estimate (g)	0.34	0.24	0.57	−1.91	2.40
*N* samples = 106	Female	Male	Unknown	Minimum	Maximum
Participants	6018	4367	638	12	2006

*Note*: Female: 54.59%, Male: 39.62%, and Unknown: 5.79%.

Abbreviation: SD, standard deviation.

### Confirmatory hypothesis testing

First, we ran the three‐level random effects model and two additional two‐level random effects models without Levels 2 (within‐study variance) and 3 (between‐study variance), respectively. Model fit indices significantly improved when three levels were included in the analysis suggesting that within‐study and between‐study variance were both statistically significant. Results revealed a moderate effect of preference for curvature (*g* = 0.39, *t* = 5.66, *p* < 0.001, 95% CI [0.25, 0.52]). Level 3 accounted for more heterogeneity (77.67%) than Level 2 (14.38%), demonstrating that in a two‐level model, heterogeneity is incorrectly attributed only to the second level (Table 2). In addition, we ran a random‐effects model without handling the dependencies between effect sizes, which produced a slightly smaller effect than the three‐level model (*g* = 0.32, *z* = 10.89, *p* < 0.001, 95% CI [0.26, 0.38]). Finally, a model with the effect sizes and variance estimates averaged across studies showed a similar effect as the original analysis (*g* = 0.39, *z* = 5.42, *p* < 0.001, 95% CI [0.25, 0.53]). Overall, these results suggest that there is a true effect of preference for curvature even when dependencies among effect sizes are accounted for.

**TABLE 2:** Fixed effects and heterogeneity estimates from the three‐level models and the two‐level models

	Fixed‐effect estimates			Heterogeneity estimates			
Models	g	SE	95% CI	T^2^_level 2_	T^2^_level 3_	I^2^_level 2_	I^2^_level 3_
Three‐level	0.39 ***	0.07	0.25, 0.52	0.04	0.24	14.38	77.67
Without Level 2	0.39 ***	0.07	0.25, 0.52	0	0.27	0	91.76
Without Level 3	0.37 ***	0.05	0.27, 0.47	0.25	0	91.26	0

Abbreviations: CI, confidence interval; SE, standard error.

*** *p* < 0.001.

When excluding influential cases based on Cook's distance (see Materials and Methods), the magnitude of preference for curvature was slightly smaller compared to the original analysis (*g* = 0.33, *t* = 5.02, *p* < 0.001, 95% CI [0.20, 0.46]). A second sensitivity analysis excluding effect sizes extracted from values represented in plots yielded a slightly larger effect compared to the original analysis (*g* = 0.42, *t* = 6.60, *p* < 0.001, 95% CI [0.30, 0.55]). Finally, a third sensitivity analysis excluding effect sizes from studies using real (three‐dimensional) stimuli showed a similar effect size compared to the original analysis (*g* = 0.36, *t* = 5.26, *p* < 0.001, 95% CI [0.22, 0.49]). Since none of these analyses revealed any major deviation from the original analysis, we retained all studies in our further exploration of the dataset.

**FIGURE 3:**
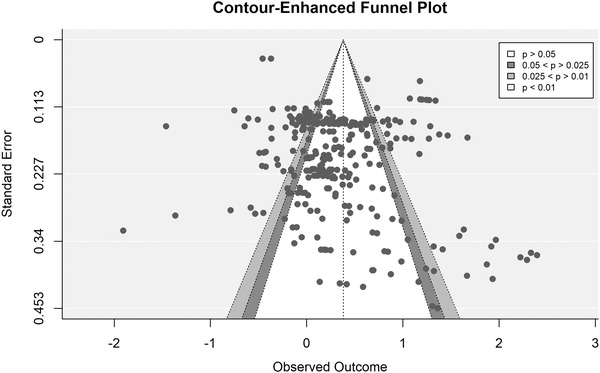
Contour‐enhanced funnel plot of the effect sizes included in the meta‐analysis. Each dot represents an effect size. The vertical line represents the overall effect of preference for curvature. Within the funnel plot, the white area shows nonsignificant effect sizes. The dark gray area shows significant effect sizes with a *p*‐value between 0.05 and 0.025. The light gray area shows effect sizes with a *p*‐value between 0.025 and 0.01. The area out of the funnel shows effect sizes with a *p*‐value smaller than 0.01.

We conducted a variant of the Egger regression test, incorporating a multi‐level meta‐analysis, to assess funnel plot asymmetry or small‐study effects while handling dependencies among effect sizes.^95^ Results indicated no evidence of a small‐study bias (*β* = 0.70, *p* = 0.32). Figure 3 shows a contour‐enhanced funnel plot of the relationship between effect size and standard error. Contour‐enhanced funnel plots make it easier to assess whether possible missing effect sizes correspond to areas of low or high statistical significance.^96^ When missing studies correspond to areas of low statistical significance, funnel plot asymmetry may be caused by publication bias. Conversely, when missing studies correspond to areas of high statistical significance, the asymmetry is less likely to be caused by publication bias. Here, visual inspection suggested that some effect sizes with small standard errors are dispersed far from the mean, especially toward the right‐side area. However, the plot does not indicate asymmetry, nor does it indicate evidence of publication bias as effect sizes are represented in both areas of low and high significance.

### Moderator analyses

The effect of preference for curvature coexists with substantial between‐study heterogeneity variance (I^2^_level 3_ = 77.67%). We ran moderator analyses with *task* (artistic, economic, semantic, hedonic, and magnetic), *stimulus type* (object, meaningless, spatial design, and symbolic design), *presentation time* (limited vs. unlimited), *measure* (continuous vs. continuous dichotomous), and *expertise* (experts, quasi‐experts, and nonexperts) as variables, in order to ascertain if these conditions account for the variability among effect sizes (see Materials and Methods for further information on how these factors were defined).

**FIGURE 4:**
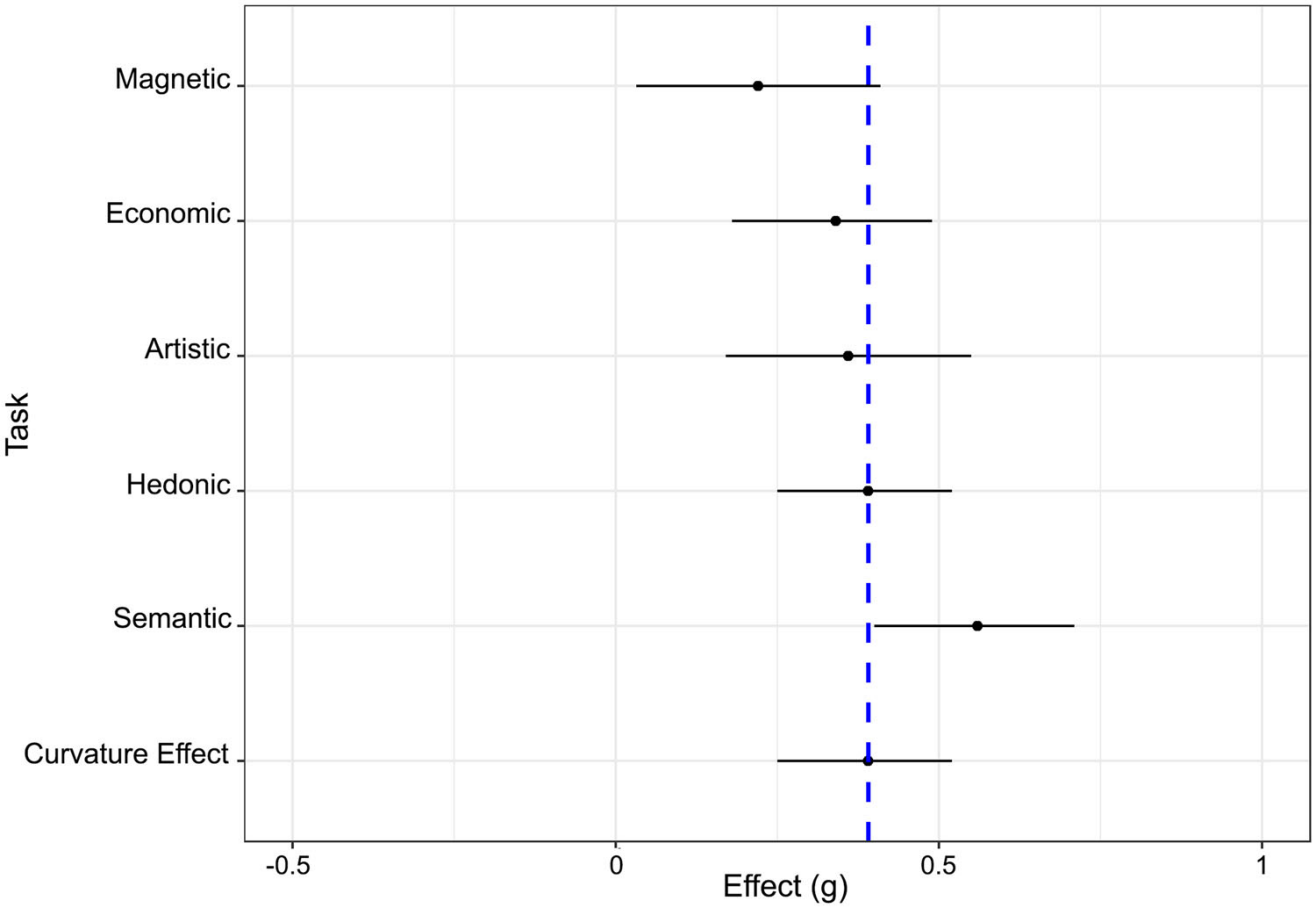
The effect of task on the magnitude of the effect of curvature. The dashed line represents the overall effect of preference for curvature. Error bars represent 95% CIs. Abbreviation: CI, confidence interval.

The effect of task was significant, Q(4) = 23.42, *p* < 0.001. The magnitude of preference for curvature was moderate‐to‐large with the semantic (*g* = 0.56, *t* = 7.05, *p* < 0.001, 95% CI [0.40, 0.71], *k* = 35) task. In turn, the effect was moderate with the hedonic (*g* = 0.39, *t* = 5.55, *p* < 0.001, 95% CI [0.25, 0.52], *k* = 184) task, and small‐to‐moderate with the artistic (*g* = 0.36, *t* = 3.75, *p* < 0.001, 95% CI [0.17, 0.55], *k* = 42) and economic (*g* = 0.34, *t* = 4.28, *p* < 0.001, 95% CI [0.18, 0.49], *k* = 31) tasks. Lastly, the effect was small with the magnetic (*g* = 0.22, *t* = 2.30, *p* = 0.022, 95% CI [0.032, 0.41], *k* = 17) task (Figure 4). Pairwise comparisons showed that the effect was significantly larger with the semantic task than with the magnetic (*g*_*diff*_ = 0.33, 95% CI [0.16, 0.50], *p* < 0.001), economic (*g*_*diff*_ = 0.22, 95% CI [0.11, 0.33], *p* < 0.001), artistic (*g*_*diff*_ = 0.19, 95% CI [0.028, 0.36], *p* = 0.022), and hedonic (*g*_*diff*_ = 0.17, 95% CI [0.078, 0.26], *p* < 0.001) tasks. Similarly, the effect was larger with the hedonic task than with the magnetic task (*g*_*diff*_ = 0.16, 95% CI [0.011, 0.31], *p* = 0.035).

The effect of stimulus type was also significant, Q(3) = 12.52, *p* = 0.0058. The magnitude of preference for curvature was moderate‐to‐large with meaningless (*g* = 0.56, *t* = 5.78, *p* < 0.001, 95% CI [0.37, 0.75], *k* = 83) stimuli and moderate with real (*g* = 0.42, *t* = 4.34, *p* < 0.001, 95% CI [0.23, 0.62], *k* = 123) stimuli. In contrast, the effect was small‐to‐moderate and nonsignificant with symbolic design (*g* = 0.30, *t* = 1.69, *p* = 0.092, *k* = 30), and small and nonsignificant with spatial design stimuli (*g* = −0.04, *t* = 0.26, *p* = 0.80, *k* = 73). Pairwise comparisons revealed that the effect with meaningless stimuli was significantly larger than the effect with spatial design stimuli (*g*_*diff*_ = 0.52, 95% CI [0.22, 0.82], *p* < 0.001). Similarly, the effect with real stimuli was larger than the effect with spatial design stimuli (*g*_*diff*_ = 0.39, 95% CI [0.062, 0.71], *p* = 0.020).

The effect of presentation time was also statistically significant, Q(1) = 15.41, *p* < 0.001. Preference for curvature was higher when stimuli were presented with limited display times (*g* = 0.75, *t* = 6.45, *p* < 0.001, 95% CI [0.52, 0.99], *k* = 50; i.e., from 84 to 7000 ms). In contrast, the effect was small‐to‐moderate with unlimited presentation times (*g* = 0.32, *t* = 4.39, *p* < 0.001, 95% CI [0.17, 0.46], *k* = 259; i.e., until response). To further examine the effect of presentation time on the cognitive processing of visual contour, we ran an additional model by considering presentation times below 1000 ms as a threshold for limited display times. In this case, the effect of presentation time did not reach statistical significance, Q(1) = 1.95, *p* = 0.16. However, results also revealed that the magnitude of preference for curvature was moderate‐to‐large with display times below or equal to 1000 ms (*g* = 0.59, *t* = 3.68, *p* < 0.001, 95% CI [0.27, 0.90], *k* = 16), while it was only moderate with display times above 1000 ms (*g* = 0.37, *t* = 5.40, *p* < 0.001, 95% CI [0.24, 0.51], *k* = 293).

We found no moderating effect of measure on curvature preference, Q(1) = 1.63, *p* = 0.20. The effect was significant with both continuous measures (*g* = 0.41, *t* = 5.71, *p* < 0.001, 95% CI [0.27, 0.55], *k* = 275) and continuous dichotomous measures (*g* = 0.27, *t* = 2.45, *p* = 0.015, 95% CI [0.054, 0.50], *k* = 34), but the difference between these measures was not significant. Finally, since there was no significant difference between experts and quasi‐expert samples, (*g*_*diff*_ = −0.13, *p* = 0.69), these categories were combined (*k* = 42) and compared to the nonexpert samples (*k* = 267). Results revealed a significant effect of expertise, Q(1) = 4.25, *p* = 0.039. While the curvature effect was moderate and significant with nonexperts (*g* = 0.40, *t* = 5.80, *p* < 0.001, 95% CI [0.27, 0.54], *k* = 267), the effect was small and nonsignificant with experts (*g* = 0.13, *t* = 0.94, *p* = 0.35, *k* = 42). From the studies working with experts, a single study recruited dental health experts and provided more than half of the expert records.^65^ In contrast, the other studies focused on experts in architecture, design, and the arts. Therefore, we repeated the model focusing on the comparison among these last experts and nonexperts. In this case, the effect of expertise was also significant, Q(1) = 8.35, *p* = 0.0039, such that it was larger with nonexperts (*g* = 0.41, 95% CI [0.27, 0.55], *p* < 0.001, *k* = 267) than with experts on architecture, design, and the arts (*g* = −0.034, *p* = 0.83, *k* = 18).

### Exploratory analyses

In addition to the preregistered protocol, we conducted some additional exploratory analyses. The moderating effect of the year of publication on curvature preference was not significant, Q(1) = 0.18, *p* = 0.67.^a^ Similarly, the moderating effect of data collection procedure was not significant, Q(1) = 0.18, *p* = 0.67. The magnitude of preference for curvature was similar when the task was carried out in person (*g* = 0.40, *t* = 5.40, *p* < 0.001, 95% CI [0.25, 0.54], *k* = 226) as when it was carried out online (*g* = 0.34, *t* = 2.72, *p* = 0.0069, 95% CI [0.094, 0.59], *k* = 83). Furthermore, the magnitude of the effect was similar regardless of whether studies used paper‐based tasks (*g* = 0.39, *t* = 2.75, *p* = 0.0064, 95% CI [0.11, 0.67], *k* = 73), computer‐based tasks (*g* = 0.34, *t* = 4.16, *p* < 0.001, 95% CI [0.18, 0.50], *k* = 131), or web‐based tasks (*g* = 0.34, *t* = 2.65, *p* = 0.0084, 95% CI [0.087, 0.59], *k* = 80). Lastly, the moderating effect of verbal terminology was not significant, Q(14) = 19.23, *p* = 0.16. The magnitude of preference for curvature was large with the terminology “curvilinear/rectilinear” (*g* = 0.90, *t* = 4.48, *p* < 0.001, 95% CI [0.51, 1.30], *k* = 30), whereas the magnitude of the effect was moderate with the terminology “round/angular” (*g* = 0.43, *t* = 2.93, *p* = 0.0037, 95% CI [0.14, 0.73], *k* = 52), and small‐to‐moderate with the terminology “curved/sharp,” (*g* = 0.40, *t* = 2.62, *p* = 0.0092, 95% CI [0.10, 0.71], *k* = 43) and “curved/angular” (*g* = 0.37, *t* = 2.54, *p* = 0.012, 95% CI [0.083, 0.66], *k* = 73).

## DISCUSSION

This meta‐analysis was conducted to compute the average effect size for visual curvature preference. The results of a three‐level random effects model revealed a Hedges’ *g* of 0.39—consistent with a medium effect size. However, they also revealed substantial between‐study heterogeneity variance, consistent with moderation effects associated with presentation time, stimulus type, expertise, and task. This finding suggests that while visual objects with a curvilinear contour are preferred to objects with an angular contour in many evaluative contexts, they are not preferred to the same extent in all contexts. Below, we discuss the possible reasons why a preference for curvature is modulated by such factors.

### Presentation time

Curvature was preferred more in evaluative contexts where responses were collected with limited presentation times than in evaluative contexts with unlimited presentation times. This observation suggests that preference for curvature emerges rather rapidly when viewing stimuli and that additional time is likely to engage top‐down processes that could serve to attenuate the effect. Basic sensory and perceptual aspects of stimuli (e.g., symmetry, contour, etc.) exert their effects as a consequence of rapid perceptual responses in early, posterior parts of the ventral visual stream, with top‐down processes representing semantics, and content occurring at later stages of the ventral visual processing pathway.^35^, ^81^, ^97^ This hypothesis is consistent with the evolutionary hypothesis that contour serves as a form of fast input to circuits that determine the relevance of visual stimuli to survival; in order for organisms to respond rapidly to potential threats, perceptual representations of angularity and curvature are relayed quickly and directly to mesocorticolimbic structures where appropriate appetitive or defensive actions can be initiated in a matter of microseconds.^98^

However, visual perception is not simply a matter of sequential forward projection of information driven by stimulation of sensory receptors.^99^, ^100^, ^101^ Expectations derived from previous experiences or task conditions are also known to modulate the way stimuli are computed by neurons involved in visual perception.^102^, ^103^ Experiments have found such expectations to influence how pleasurable an object is experienced to be,^104^, ^105^ with prior preferences biasing evoked neural activity in both perceptual and valuation regions.^106^, ^107^, ^108^ It is, therefore, conceivable that a higher preference for curvilinear stimuli observed during evaluation events where stimuli are presented only for a brief time period reflect predictive coding as much as bottom‐up driven processing. It will be important for future studies to clarify how computational mechanisms involved in visual liking unfold over different temporal time scales.

### Stimulus type

Preference for curvature was also shown to be stronger for real and imaginary objects than for spatial designs and/or symbols. This observation could be explained by several factors. In terms of their affordances—defined as the actions or uses that they enable^109^—contour might be a more salient and relevant feature for objects (whether real or imaginary) than spaces or symbols. In other words, whether an object's form is curvilinear or angular might play a more important role in our choices to interact with them than might be the case with spaces and symbols. Another possibility might be mere exposure.^110^ Specifically, we may encounter curvilinear and angular objects more frequently than we do curvilinear and angular spaces and symbols, and as such develop greater levels of processing fluency in evaluating them.^111^, ^112^ In this sense, the larger effect size for contour for objects compared to spaces and symbols could reflect our greater ability to distill and appraise the sensory and perceptual features of the former type of stimuli. Indeed, the same could be true for imaginary objects given that they too represent object‐like features when used as stimuli in experimental studies, including boundaries, contrast, and symmetry, among others.

### Expertise

Participants’ expertise also affected preference for curvature, with a stronger effect in studies recruiting nonexpert than expert participants. However, this result should be interpreted carefully because out of the 61 studies included in the meta‐analysis, only six recruited expert participants. Moreover, while our findings suggest that expertise modulates people's sensitivity and preference for curvature, it is likely that this is true only when the evaluated objects are specific to the participant's field of expertise.^32^

### Task

Finally, evaluative task conditions also moderated the size of the effect of curvature. Recall that across studies researchers had asked participants to evaluate curvilinear and angular stimuli using diverse evaluative anchors (e.g., approach/avoidance, attractiveness, beauty, comfortableness, valence, liking, price expectation, wanting, etc.). Different evaluative anchors evoke computational mechanisms associated with hedonic valuation to different degrees,^113^, ^114^ presumably because evaluative anchors, such as *beauty* or *liking*, prompt participants to evaluate stimuli according to different evaluative target dimensions.^115^, ^116^

To examine in greater detail how the use of different evaluative anchors affects curvature preference, we grouped the included tasks into five bins (semantic, artistic, economic, magnetic, and hedonic). The effect of preference for curvature was largest for evaluations that used semantic tasks, registering a significant difference compared to the other tasks. Semantic tasks were those that involved categorizations based on the semantic differential scales of the evaluative dimension used to measure the value of an object,^63^ such as good/bad, positive/negative, aggressive/peaceful, and so on. This effect might indicate that contour could have a strong semantic association with the dimensions under consideration.^12^, ^19^ In other words, there could be a strong semantic association between curvilinear and angular forms and the opposing poles of those dimensions. Another possibility might be that compared to semantic tasks, other task conditions bring additional contextual and individual‐differences factors into play that could serve to weaken the effect of contour on choice. For example, judging whether a face is attractive or not (i.e., a hedonic task) necessitates that the participant activates a mental representation of attractiveness which might vary considerably across individuals. In turn, this variation might interact with the task. Similarly, deciding how much one would like to pay for an object (i.e., an economic task) requires the participant to activate a mental representation of monetary value which might again vary across individuals based on background factors, such as socioeconomic status. This line of reasoning suggests that one is more likely to observe a strong preference for curvature if the evaluative task involves a relatively direct evaluation of the stimulus along a dimension used to measure its value.

### What is the cause of curvature preference?

Together, our results demonstrate that preference for visual curvature is influenced by factors other than perceptual information signaling contour shape. This finding highlights the need for future research that describes in more detail the computational mechanisms that determine individual liking responses to visual objects with curvilinear contour. The current consensus among neuroscientists holds that liking and disliking for sensory stimuli occur as a function of information transfer from sensory systems to the mesocorticolimbic reward circuit.^117^, ^118^ Liking and disliking outcomes appear to be determined by the state and intensity of pleasure and displeasure elicited in response to a given stimulus.^119^, ^120^ The manner in which nuclei that encode pleasure and displeasure become engaged by information from sensory systems is often modulated by individual differences in how brains are functionally and structurally organized, as well as by the contextual conditions under which the stimulus is being appraised.^115^, ^121^ We believe that a similar process may be at play for preference for curvature in the form of a loop that connects the sensory cortices that exhibit sensitivity to the perception of contour to regions within the brain that underlie the valuation of stimuli. Consistent with this idea, several meta‐analyses have revealed that the aesthetic evaluation of objects in the visual domain engages regions within sensory and perceptual cortices, as well as regions of the brain that underlie the processing of reward.^122^, ^123^, ^124^ What remains unknown are the specific mechanisms that underlie the transfer of information from the sensory and perceptual cortices to the reward regions, which eventually leads to an evaluative appraisal of the object under consideration.

A recent study by Yue et al.^125^ has made important strides in this regard by demonstrating that patches of neurons located in bilateral V3 and V4, as well as in the lateral occipitotemporal cortex (LOC) and fusiform gyrus (FG), respond preferentially to curvilinear contour. This result suggests that both regions in the earlier and later parts of the ventral visual system are involved in representing how curvilinear or angular an object's contour is perceived to be. However, it remains unknown how neural activity evoked by this network of neurons affects activity in other neural systems, including the reward circuitry. Fortuitously, analytic methods involving fMRI data (e.g., dynamic causal modeling) exist that can test how patterns of connectivity between V3, V4, LOC, FG, and other regions of interest affect liking outcomes for stimuli varying in contour shape, and we expect this endeavor to be a likely focus of research in neuroscientific studies on the effect of contour on hedonic valuation.

### Limitations and future directions

We focused only on studies employing continuous measures (i.e., rating scales or two‐alternative responses) and were not able to compute the effect sizes from all the studies meeting eligibility criteria because of insufficient data for their calculation. When we computed the average effect size of the smaller set of studies employing dependent dichotomous measures,^21^, ^30^, ^33^, ^40^, ^47^, ^48^, ^49^, ^50^, ^51^, ^52^, ^53^ results indicated an odds ratio of 2.13—consistent with a small effect size.^126^ Nevertheless, this meta‐analysis comprises a relevant set of studies that investigated the effect of visual curvature under various conditions providing an estimate of the true effect of preference for visual curvature.

Our findings also provide some implications for applied research on contour preference. As reviewed here, many studies from multidisciplinary fields have investigated preference for visual curvature with an increasing number of experiments from applied domains, such as advertising, marketing, packaging, interior design, and security perception, among others.^10^ Thus, these domains could benefit from our results by targeting people's preferences or environmental perceptions. For example, marketers could employ specific evaluative anchors to advertise hedonic or utilitarian products with different contour types; or artists could gain insights into how the audience's shape preferences may vary depending on their previous experience in design, architecture, or the arts. Moreover, our findings could also benefit the design of ecological and friendlier environments, which may foster people's well‐being and inclusiveness.^37^ Importantly, this meta‐analysis also has isolated conditions under which the strength of preference for visual curvature is likely to be maximal. Thus, future research in vision and visual neuroscience could benefit by establishing an empirical benchmark for the strength of this effect (i.e., Hedges’ *g* of 0.39) before examining its strength based on more rigorous manipulations.

Our moderator analyses demonstrated the presence of notable variance that is left unaccounted for, which could be explained by other variables not considered in our analysis.^127^ Part of this difficulty lies in the heterogeneity with which the same core constructs have been conceptualized and measured in this literature. For example, researchers have long suspected that relevant domain expertise might impact one's sensitivity to and preference for curvature. However, whereas some studies have evaluated the influence of expertise by recruiting expert and nonexpert participants,^13^, ^34^, ^37^, ^65^, ^79^, ^128^ others have assessed expertise as a continuous variable/trait via self‐reported questionnaires.^14^, ^15^, ^32^, ^36^, ^50^, ^51^, ^81^, ^90^, ^129^ Furthermore, when recruiting experts, some studies have recruited true experts (e.g., Ref. 34), whereas others have relied on quasi‐experts (e.g., Ref. 37)—despite well‐established differences between true experts (i.e., professionals with formal training working in a field) and quasi‐experts (i.e., apprentices or graduate students in a field) in relative familiarity with a domain. We suspect that future reviews and meta‐analyses of this growing literature will examine the literature in other ways than we have here, perhaps with sharper conceptual and operational definitions of key constructs under investigation, including expertise.

We also believe that the next frontier in this field will likely involve two advances. First, currently, there is no computationally derived consensus measure for quantifying the degree of curvature involving visual stimuli. The discovery of such a mathematical algorithm will be useful as a standardization tool, enabling comparison of what is meant by curvature across stimulus categories (e.g., objects, scenes, and artworks). Second, the neurobiological mechanisms that give rise to a preference for contour have yet to be unearthed. Building on recent research that has revealed regions in the occipital and temporal cortices that are sensitive to contour, we believe that the next frontier in this line of research involves identifying the neurobiological mechanisms that give rise to a preference for contour—connecting sensory perception to hedonic valuation.

## CONCLUSIONS

Substantial empirical evidence gathered over the last century shows that people prefer curvature in the visual domain across many tasks and contexts. Because preference for curvature has also been documented across ages, cultures, and species, it has come to be viewed as potentially a universal phenomenon in the visual domain. On the other hand, it is also clear that the occurrence and strength of preference for curvature are influenced by individual differences and contextual factors. Here, we conducted a meta‐analysis of the empirical research on contour to calculate the strength of the effect of preference for curvature in the visual domain. The results of a three‐level random effects model revealed a Hedges’ *g* of 0.39—consistent with a medium effect size. This effect was moderated by presentation time, stimulus type, task, and expertise. Together, our results show that people's preference for curvature in the visual domain is general and common, though not universal and invariant.

## AUTHOR CONTRIBUTIONS

O.V., M.S., M.N., P.J.S., and E.M. conceived the idea. E.G.C., G.B.C., and E.M. designed the study protocol and the data analysis plan. E.G.C., O.V., G.B.C., and E.M. performed the literature searches. E.G.C., G.B.C., and E.M. screened the studies and analyzed the data. E.G.C., O.V., M.S., and E.M. interpreted the results. E.G.C., O.V., M.S., M.N., and P.J.S. drafted the manuscript. All authors edited, revised, and approved the final version of the manuscript.

## COMPETING INTERESTS

The authors declare that there are no competing interests.

### PEER REVIEW

The peer review history for this article is available at https://publons.com/publon/10.1111/nyas.14919.

## Supporting information

Supplementary Information

## Footnotes

a This analysis, intended to test for the possible effects of societal and cultural changes in taste, was suggested by Melanie Wald–Fuhrmann.
