# Supplementary material for: How universal is preference for visual curvature? A systematic review and meta‐analysis
Source: Ann N Y Acad Sci. 2022 Oct 26;1518(1):151–65. doi: 10.1111/nyas.14919 (PMC10091794; doi:10.1111/nyas.14919)
Supplement: Supplementary file 1 — Supplementary Information [file NYAS-1518-151-s001.docx]

**Automated Database Search**

**Date:** 21/02/2021

**EBSCOHost – PsycINFO**

**Title (215 records):** TI ("curvature" OR "curvilinear" OR "smooth" OR "round" OR "curved" OR "sharp-angled" OR "sharp" OR "angular" OR "straight" OR "rectilinear") AND TI ("contour" OR "shape") AND TI ("aesthetic" OR "preference" OR "liking" OR "beauty").

**Keywords (4 records):** KW ("curvature" OR "curvilinear" OR "smooth" OR "round" OR "curved" OR "sharp-angled" OR "sharp" OR "angular" OR "straight" OR "rectilinear") AND KW ("contour" OR "shape") AND KW ("aesthetic" OR "preference" OR "liking" OR "beauty").

**Abstract (5 records)**: AB ("curvature" OR "curvilinear" OR "smooth" OR "round" OR "curved" OR "sharp-angled" OR "sharp" OR "angular" OR "straight" OR "rectilinear") AND AB ("contour" OR "shape") AND AB ("aesthetic" OR "preference" OR "liking" OR "beauty").

**Total records included:** 215 records (9 records were repeated).

**PubMed**

**Title/Abstract (325 records):** (("curvature"[Title/Abstract] OR "curvilinear"[Title/Abstract] OR "smooth"[Title/Abstract] OR "round"[Title/Abstract] OR "curved"[Title/Abstract] OR "sharp-angled"[Title/Abstract] OR "sharp"[Title/Abstract] OR "angular"[Title/Abstract] OR "straight"[Title/Abstract] OR "rectilinear"[Title/Abstract]) AND ("contour"[Title/Abstract] OR "shape"[Title/Abstract])) AND ("aesthetic"[Title/Abstract] OR "preference"[Title/Abstract] OR "liking"[Title/Abstract] OR "beauty"[Title/Abstract]).

**Web of Science**

**Title (31 records)**: TI=(curvature* OR curvilinear* OR smooth* OR round* OR curved) AND TI=(contour* OR shape) AND TI=(aesthetic* OR preference* OR liking* OR beauty).

**Abstract/Title (115 records)**: AB=(curvature* OR curvilinear* OR smooth* OR round* OR curved) AND TI=(contour* OR shape) AND TI=(aesthetic* OR preference* OR liking* OR beauty).

**Keyword Plus/Title (10 records):** KP=(curvature* OR curvilinear* OR smooth* OR round* OR curved) AND TI=(contour* OR shape) AND TI=(aesthetic* OR preference* OR liking* OR beauty).

**Total records included:** 156 records

**Total Database Search:** N = 696 records.

**Automated Journal Search**

**Date:** 21/02/2021

Manual journal search within six relevant journals in the Empirical Aesthetics field.

**Psychology of the Aesthetics Creativity and The Arts:** 6 records.

**Empirical Studies of the Arts, i-Perception and Perception (SAGE journals).**

**Title (1 record):** for [[Title curvature] OR [Title curvilinear] OR [Title smooth] OR [Title round] OR [Title curved] OR [Title sharp-angled] OR [Title sharp] OR [Title angular] OR [Title rectilinear] OR [Title straight]] AND [[Title contour] OR [Title shape]] AND [[Title aesthetic] OR [Title preference] OR [Title liking] OR [Title beauty]]

**Abstract (34 records):** For [[Abstract curvature] OR [Abstract curvilinear] OR [Abstract smooth] OR [Abstract round] OR [Abstract curved] OR [Abstract sharp-angled] OR [Abstract sharp] OR [Abstract angular] OR [Abstract rectilinear] OR [Abstract straight]] AND [[Abstract contour] OR [Abstract shape]] AND [[Abstract aesthetic] OR [Abstract preference] OR [Abstract liking] OR [Abstract beauty]]

**Keywords (0 records)**: For [[Keyword curvature] OR [Keyword curvilinear] OR [Keyword smooth] OR [Keyword round] OR [Keyword curved] OR [Keyword sharp-angled] OR [Keyword sharp] OR [Keyword angular] OR [Keyword rectilinear] OR [Keyword straight]] AND [[Keyword contour] OR [Keyword shape]] AND [[Keyword aesthetic] OR [Keyword preference] OR [Keyword liking] OR [Keyword beauty]]

**Total SAGE Journals:** 34 records (1 record was repeated).

**Acta Psychologica:** 33 records.

**British Journal of Psychology:** 4 records.

**Abstract:** curvature OR curvilinear OR smooth OR round OR curved OR sharp-angled OR sharp OR angular OR rectilinear OR straight" in Abstract and "contour OR shape" in Abstract and "aesthetic OR preference OR liking OR beauty" in Abstract published in "British Journal of Psychology"

**Title:** curvature OR curvilinear OR smooth OR round OR curved OR sharp-angled OR sharp OR angular OR rectilinear OR straight" in Title and "contour OR shape" in Title and "aesthetic OR preference OR liking OR beauty" in Title published in "British Journal of Psychology"

**Total Journal Search:** N = 77 records.

**Flow diagram summary** (773 records)

Studies included after removing **Duplicates**: N = 612 (161 excluded).

Studies Included based on **Title**: N = 132 (PubMed 39 (325), Journals 18 (77), EBSCOHOST and WOS 75 (372)).

Studies included based on **abstract**: N = 64 (PubMed 28, EBSCOHOST and WOS 27, Journal search 9).

Studies included based on **full-text**: N = 30.

Additional records included from **manual search** from citation and reference lists of the included studies

- Studies included from reference lists of the studies already included: N = 27.
- Studies included from citation lists of the studies already included: N = 24.

Studies included after critical appraisal and data availability: N = 61.
